# Extracellular Administration of BCL2 Protein Reduces Apoptosis and Improves Survival in a Murine Model of Sepsis

**DOI:** 10.1371/journal.pone.0014729

**Published:** 2011-02-24

**Authors:** Akiko Iwata, R. Angelo de Claro, Vicki L. Morgan-Stevenson, Joan C. Tupper, Barbara R. Schwartz, Li Liu, Xiaodong Zhu, Katherine C. Jordan, Robert K. Winn, John M. Harlan

**Affiliations:** 1 Department of Surgery, University of Washington, Seattle, Washington, United States of America; 2 Department of Medicine, University of Washington, Seattle, Washington, United States of America; Singapore Immunology Network, Singapore

## Abstract

**Background:**

Severe sepsis and septic shock are major causes of morbidity and mortality worldwide. In experimental sepsis there is prominent apoptosis of various cell types, and genetic manipulation of death and survival pathways has been shown to modulate organ injury and survival.

**Methodology/Principal Findings:**

We investigated the effect of extracellular administration of two anti-apoptotic members of the BCL2 (B-cell lymphoma 2) family of intracellular regulators of cell death in a murine model of sepsis induced by cecal ligation and puncture (CLP). We show that intraperitoneal injection of picomole range doses of recombinant human (rh) BCL2 or rhBCL2A1 protein markedly improved survival as assessed by surrogate markers of death. Treatment with rhBCL2 or rhBCL2A1 protein significantly reduced the number of apoptotic cells in the intestine and heart following CLP, and this was accompanied by increased expression of endogenous mouse BCL2 protein. Further, mice treated with rhBCL2A1 protein showed an increase in the total number of neutrophils in the peritoneum following CLP with reduced neutrophil apoptosis. Finally, although neither BCL2 nor BCL2A1 are a direct TLR2 ligand, TLR2-null mice were not protected by rhBCL2A1 protein, indicating that TLR2 signaling was required for the protective activity of extracellularly adminsitered BCL2A1 protein *in vivo*.

**Conclusions/Significance:**

Treatment with rhBCL2A1 or rhBCL2 protein protects mice from sepsis by reducing apoptosis in multiple target tissues, demonstrating an unexpected, potent activity of extracellularly administered BCL2 BH4-domain proteins.

## Introduction

Severe sepsis and its major complications of multiple organ dysfunction and shock are major causes of morbidity and mortality worldwide. Although there have been substantial improvements in antimicrobial therapy and supportive critical care, severe sepsis remains the leading cause of death among hospitalized patients in non-coronary intensive care units. Unfortunately, decades of clinical trials with a variety of drug candidates have largely failed to reduce mortality [1], [2]. Consequently, there is an urgent need to understand better the pathophysiology of severe sepsis and identify new therapeutic targets.

One of the hallmarks of experimental sepsis is increased apoptosis of a number of cell types. Although apoptosis of immune cells has been investigated most extensively [3], [4], there is also apoptosis of other cell types [5], including endothelial cells [6], [7], neutrophils [8], hepatocytes [9], cardiomyocytes [10], and intestinal epithelial cells [11]. Moreover, genetic manipulation of cell death and survival pathways has marked effects in murine models of sepsis (e.g., [12]). These observations suggest that pharmacological inhibition of apoptosis by blocking cell death pathways or promoting cell survival pathways could provide a new therapeutic approach to sepsis in man [5].

BCL2 and BCL2A1 (A1, Bfl-1) are both anti-apoptotic members of the larger BCL2 family of intracellular proteins. Transgenic over-expression of BCL2 in T-cells and B-cells [13], intestinal epithelium [14] and myeloid cells [15] all resulted in a decrease in apoptosis in those cell types and improved survival in septic mice. Thus, increased intracellular BCL2 in multiple lineages is able to confer protection in experimental sepsis.

There is growing evidence that some proteins with well-defined intracellular functions can exert new activities when released extracellularly, engaging the innate immune system to act as danger signals to promote host defense or repair, i.e., alarmins [16] or danger-associated molecular patterns (DAMPs) [17], many of which are hydrophobic molecules [18]. For example, high mobility group box 1 (HMGB1) is a nucleoprotein that is released extracellularly when cells undergo necrosis or, in some cell types, by a non-canonical secretory pathway. High levels of HMGB1 accumulate in animals [19] and humans with severe sepsis [20], [21]. Administration of antibodies against HMGB1 results in increased survival and protection against organ damage in septic animals [22]. However, even for this prototypic DAMP the mechanism(s) by which it acts *in vivo* remains uncertain as highly purified HMGB1 lacks significant activity *in vitro* [23]-[25]. Recently, we reported that the intracellular BCL2 and BCL2A1 proteins and peptides derived from them that contain the BH4-domain function as cytoprotective DAMPs, conferring tissue protection when administered extracellularly [26]. At very low, cytokine-like doses extracellular administration of rhBCL2 or rhBCL2A1 protein or peptides containing the BH4 domain of BCL2 or BCL2A1 reduced apoptosis and tissue damage in stringent murine models of hind limb and myocardial ischemia-reperfusion injury. Further, in preliminary studies rhBCL2A1 protein also reduced hypertrophy and fibrosis in pressure overload-induced heart failure [26]. Here, we show that extracellular administration of rhBCL2 or rhBCL2A1 protein also provides striking protection in experimental sepsis induced by CLP, and that this protective effect is associated with marked reduction of apoptosis in heart, intestine, and peritoneal neutrophils. These studies extend the range of experimental models of tissue and organ injury in which BCL2 BH4-domain proteins exert a protective effect, further highlighting their novel and potent extracellular activity.

## Results

### Treatment with extracellular rhBCL2A1 or rhBCL2 protein improves survival after CLP

**Figure 1:**
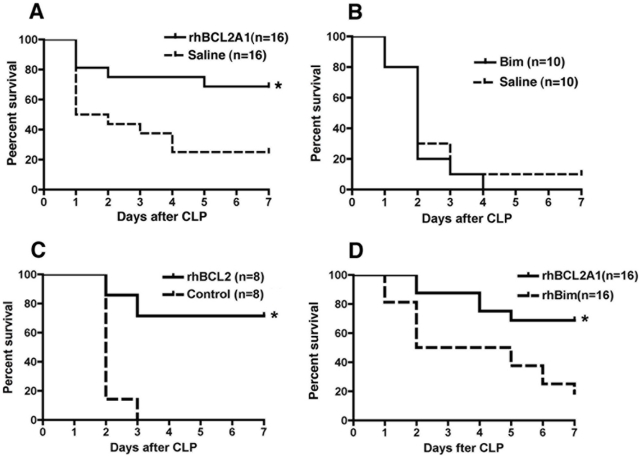
Treatment with rhBCL2A1 or rhBCL2 protein improves survival following CLP. Mice were subjected to CLP and followed for 7 days. An assessment table was used as a surrogate marker for death and the animals euthanized according to approved criteria. (A) Mice were treated by i.p. injection of 1 µg rhBCL2A1 or saline 18 hours prior to CLP and then every 12 hours for 3 consecutive days. Treatment with rhBCL2A1 conferred significant protection compared to saline. *p = 0.012. (B) Mice were treated by i.p. injection of 1 µg rhBim or saline 18 hours prior to CLP and then every 12 hours for 3 consecutive days. Treatment with rhBim did not improve survival compared to saline. (C) Mice were treated by i.p. injection of 1 µg rhBCL2 or 0.5 µg of rhUbiquitin at 18 hours prior to CLP, at time of CLP, and then every 12 hours for 3 consecutive days. Treatment with rhBCL2 conferred significant protection compared to rhUbiquitin *p = 0.003. (D) Mice were treated by i.p. injection of 1 µg rhBCL2A1 or rhBim at 18 hours prior to CLP and then every 12 hours for 3 consecutive days. Treatment with rhBCL2A1 conferred significant protection compared to rhBim. *p = 0.005.

Mice were subjected to CLP and followed for 7 days as described in Methods. An assessment table was used as a surrogate marker for death and the animals euthanized according to approved criteria. In the initial experiments, mice were treated with 1 µg (∼40 picomoles) of rhBCL2A1 or saline vehicle at 18 hours prior to CLP and then every 12 hours for 3 consecutive days for a total of eight doses. Treatment with the BH4-domain protein, rhBCL2A1, was protective (Fig. 1A), whereas treatment with the BH3-domain protein, rhBim, was not (Fig. 1B). In separate experiments, rhBCL2A1 and rhBCL2 were compared directly with other recombinant proteins, which had been prepared similarly in *E. coli* and were also his-tagged. Treatment with the anti-apoptotic BH4-domain BCL2 proteins conferred a significant increase in survival, compared with treatment with the control recombinant proteins, rhUbiquitin (Fig. 1C) or rhBim (Fig. 1D). Consequently, rhBim was used as control in most experiments.

### Treatment with extracellular rhBCL2 or rhBCL2A1 reduces apoptosis after CLP

**Figure 2:**
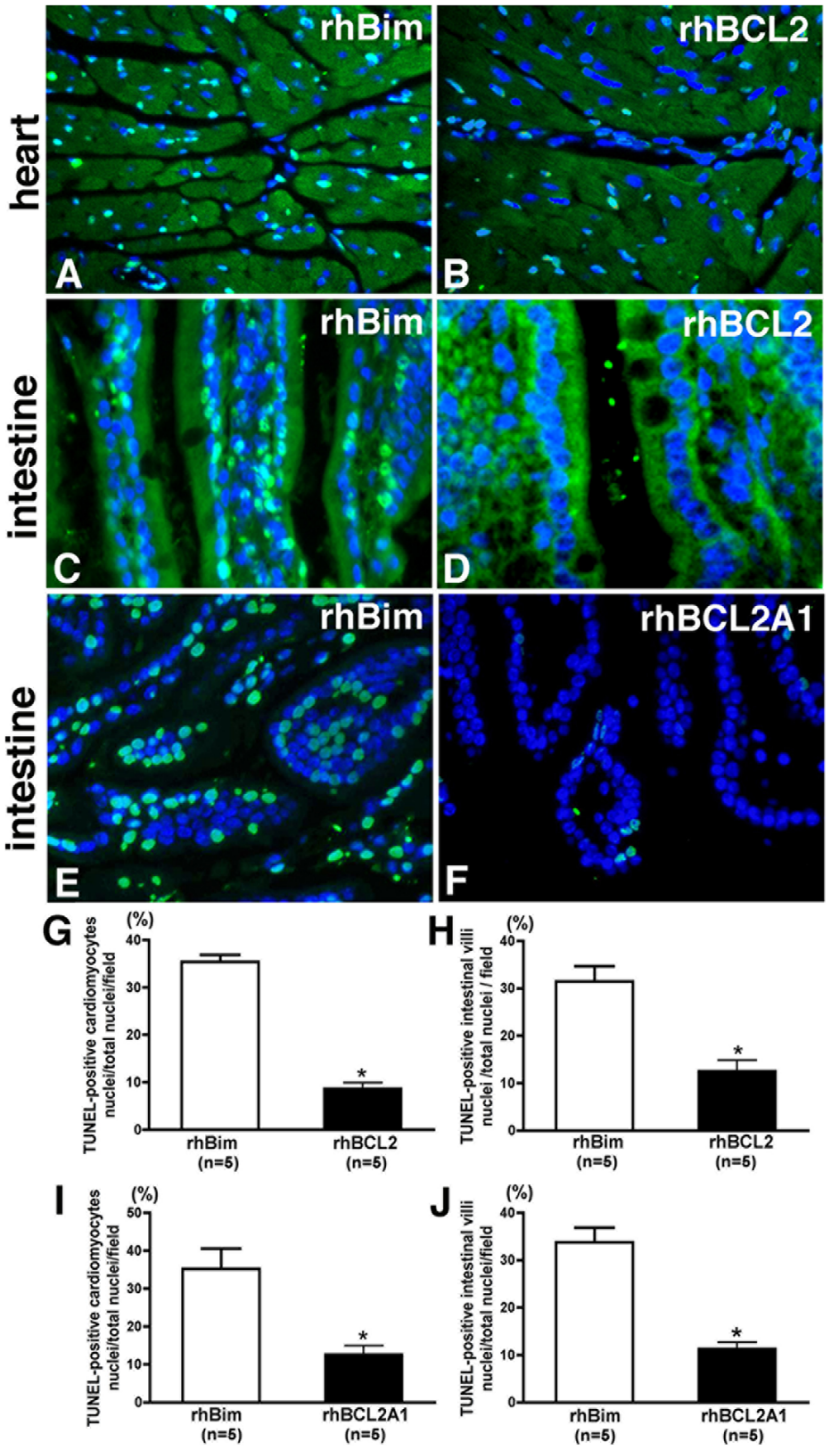
Treatment with rhBCL2 or rhBCL2A1 reduces apoptosis in intestine and myocardium following CLP. Animals were treated with a single i.p. injection of 1 µg rhBim or rhBCL2 or rhBCL2A1 at 18 hours prior to CLP. Tissues were removed and processed at 24 hours following CLP. In the photomicrograph (A-F) TUNEL-positive cells are labeled with FITC (green) and nuclei are labeled with DAPI (blue). Panels A and B shows myocardium from mice treated with rhBim (A) or rhBCL2 (B). Panels C-F show intestinal villi from mice treated of rhBim (C, E), rhBCL2 (D) or rhBCL2A1 (F). The graph shows quantitation of TUNEL-staining in cardiomyocytes (G, I) or intestine (H, J) following treatment with rhBim or rhBCL2 (G, H) or rhBCL2A1 (I, J). The number of TUNEL-positive cells in mice treated with rhBim was significantly greater than in mice treated with rhBCL2 or rhBCL2A1. *p<0.05.

**Figure 3:**
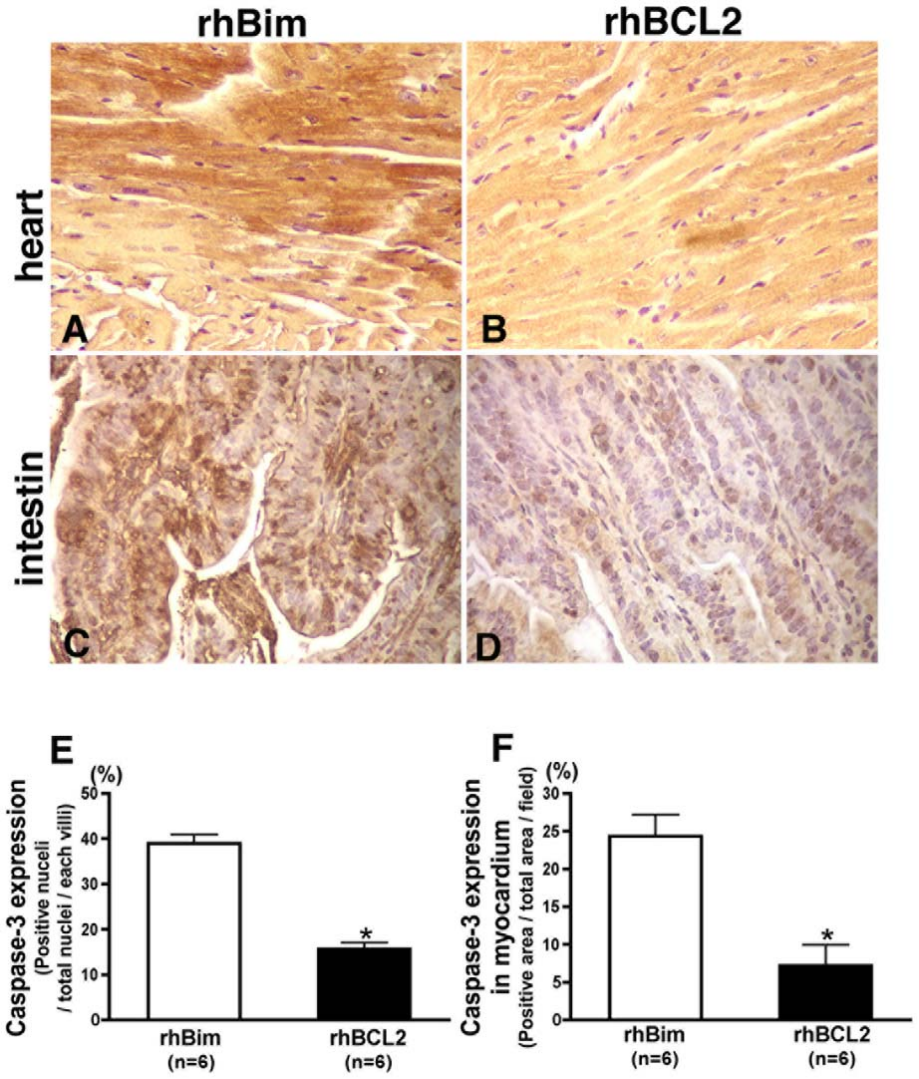
Treatment with rhBCL2 protein reduces cleaved caspase-3 in heart and intestine of mice following CLP. Mice were treated with a single i.p. injection of 1 µg rhBim (A, C) or 1 µg rhBCL2 (B, D) at 18 hours prior to CLP. Heart (A, B) or intestine (C, D) was stained for cleaved caspase with 3, 3′-diaminobenzidine (brown). Nuclei were stained with hematoxylin (purple). Original magnification is 400×. Panels E and F show quantitation of cleaved caspase-3 expression in myocardium (E) and intestinal villi (F) in mice treated with rhBim compared to those treated with rhBCL2. *p<0.05.

As compared to mice treated with rhBim (Fig. 2A, C), treatment with rhBCL2 (Fig. 2B, D) reduced the number of TUNEL-positive cells detected at 24 hours after CLP in the heart by 76% (Fig. 2G) and in the intestine by 58% (Fig. 2H). Likewise, treatment with rhBCL2A1 significantly reduced the number of TUNEL-positive cells in the heart (Fig. 2I) and the intestine (Fig. 2F, J). Furthermore, mice treated with rhBCL2 protein had a significant reduction in cleaved caspase-3 expression following CLP in the heart (Fig. 3B, F) and intestine (Fig. 3D, E) compared to mice treated with rhBim (Fig. 3A, C, E, F). Taken together, these data indicate that treatment with rhBCL2 or rhBCL2A1 protein reduces sepsis-induced apoptosis in multiple target organs.

### Endogenous mouse BCL2 protein is up-regulated in the hearts and intestine of rhBCL2A1-treated mice

**Figure 4:**
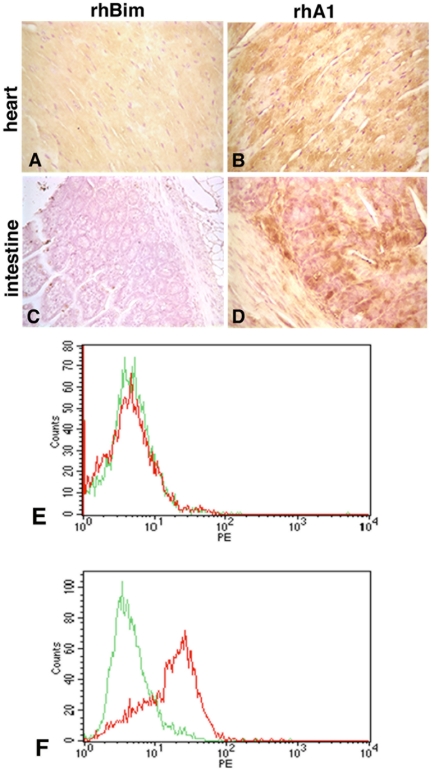
Treatment with rhBCL2A1 increases expression of endogenous BCL2 following CLP. Mice were treated with a single i.p. injection of 1 µg rhBim (A, C) or rhBCL2A1 (B, D) at 18 hours prior to CLP. Heart (A, B) and intestine (C, D) were examined at 24 hours following CLP. Endogenous mouse BCL2 protein was detected by immuno-staining with rabbit anti-mouse BCL2 antibody detected with 3, 3′-diaminobenzidine (brown) and the nuclei were stained with hematoxylin (purple). Representative FACS analysis of endogenous BCL2 expression in cardiomyocytes from mice treated as above with rhBim (E) or rhBCL2A1 (F). Green line represents isotype control and red line represents anti-mouse BCL2 antibody.

We next determined whether treatment with rhBCL2A1 altered expression of endogenous BCL2. Using an anti-mouse BCL2 antibody that does not cross-react with human BCL2, we found that mice treated with rhBCL2A1 (Fig. 4B, D) showed increased expression of endogenous mouse BCL2 protein in both the heart muscle and intestine, as compared to mice that were untreated or treated with rhBim (Fig. 4A, C). We confirmed the up-regulation of endogenous mouse BCL2 protein using flow cytometry analysis. Cardiomyocytes from mice treated with rhBCL2A1 showed an increase in endogenous BCL2 expression 24 hours after CLP (Fig. 4E, F). Taken together, these data suggest that protection may be conferred in some tissues by up-regulation of endogenous BCL2 in the stressed tissue.

### Cell populations and cytokines in peritoneum and peripheral blood

Peripheral blood leukocyte counts were analyzed following CLP. Mice were treated with a single i.p. injection of 1 µg rhBCL2A1 or 1 µg of rhBim at 18 hours prior to CLP. Animals were euthanized at 24 hours following CLP and whole blood was analyzed. Peripheral blood leukocyte counts and neutrophil counts were similar in rhBCL2-treated and rhBim-treated animals (total leukocytes were 2.184×10^6^ +/− 0.3336 SEM in rhBim-treated and 1.993×10^6^ +/− 0.4202 SEM in rhBCL2A1-treated; total neutrophils were 0.8356×10^6^ +/− 0.8356 SEM in rhBim-treated and 0.7789×10^6^ +/− 0.1221 SEM in rhBCL2A1-treated).

**Figure 5:**
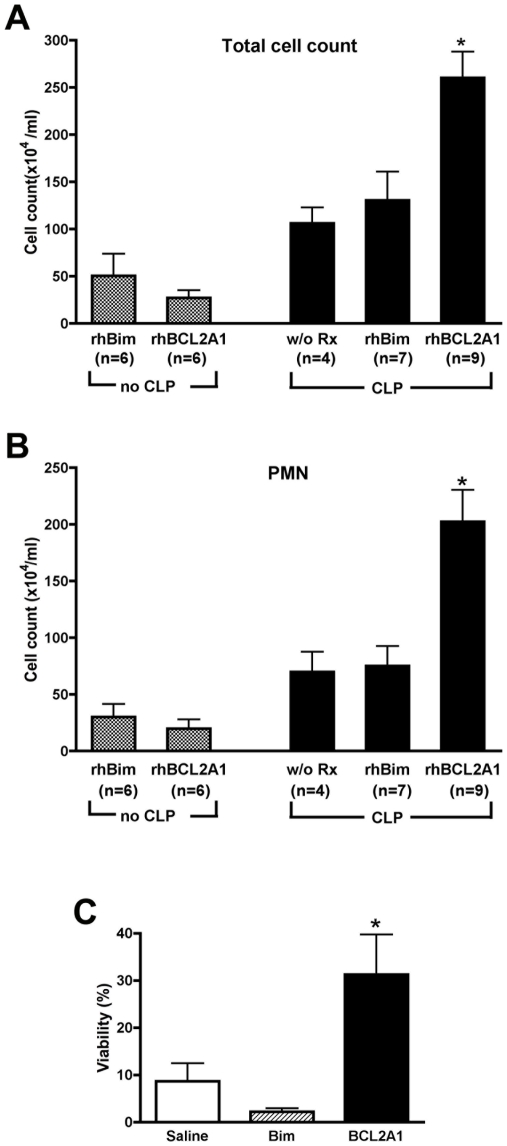
Treatment with rhBCL2A1 increases neutrophils in peritoneum following CLP. Mice were treated once by i.p. injection with 1 µg of rhBCL2A1 or rhBim at 18 hours prior to CLP. Peritoneal lavage fluid was collected at 24 hours following CLP. Panels A and B show the total number of leukocytes and neutrophils (PMNs) in lavage fluid. Mice treated with rhBCL2A1 showed an increase in peritoneal PMNs following CLP as compared to mice that did not receive CLP or mice that were treated with rhBim. Panel C shows the viability of peritoneal neutrophils at 24 hours following CLP as assessed by staining with annexin V and 7-amino-actinomycin D. Treatment with rhBCL2 significantly increased the number of viable neutrophils compared to treatment with saline or rhBCL2A1. *p<0.01.

In separate experiments, leukocyte populations in peritoneal lavage were evaluated. Mice received rhBim, rhBCL2A1, or no treatment followed 18 hours later by CLP. Additionally, some mice received either rhBim or rhBCL2A1, but did not have CLP. In those animals not subjected to CLP, there was no significant accumulation of total leukocytes or neutrophils in animals treated with rhBCL2A1 (Fig. 5 A, B). In contrast, in animals subjected to CLP there was a significant increase in the total number of leukocytes in the peritoneal lavage fluid in mice treated with rhBCL2A1 as compared to the other treatment groups (Fig. 5A). This increase in total cells was due specifically to an increase in neutrophils in the peritoneal lavage fluid (Fig. 5B). We did not observe any changes in circulating neutrophil counts, and G-CSF levels in peritoneal fluid and G-CSF mRNA expression in spleen (Fig. S1) were similar in the rhBCL2A1-treated mice compared to rhBim-treated mice following CLP, making it less likely that the increase in peritoneal neutrophils following CLP was due solely to increased production. However, annexin V-staining of peritoneal cells showed that there were more viable neutrophils in the peritoneal lavage of rhBCL2A1-treated animals following CLP (Fig. 5C), consistent with a reduction in apoptosis.

We determined the generation of reactive oxygen species by peritoneal neutrophils as determined by flow cytometry using dihydrorhodamine-123 to detect intracellular ROS. At 24 hours post-CLP there was no difference in ROS generation by unstimulated neutrophils between saline-treated mice and rhBCL2A1-treated mice (mean channel fluorescence: 8.07±4.5 in saline versus 7.06±2.2 in rhBCL2A1; means±SD of 3 mice). Similarly, there was no difference between saline-treated and rhBCL2A1-treated following stimulation by phorbol myristate acetate (mean channel fluorescence: 17.4±4.1 in saline versus 25.7±9.0 in rhBCL2A1; means±SD of 3 mice).

We also assayed the levels of 20 different cytokines, chemokines, or growth factors in the peritoneal lavage fluid. Despite the marked improvement in survival with treatment with BCL2A1, there were no significant differences between levels in rhBCL2A1- and rhBim-treated animals (Table S1).

### Protection by rhBCL2A1 requires TLR2 signaling

**Figure 6:**
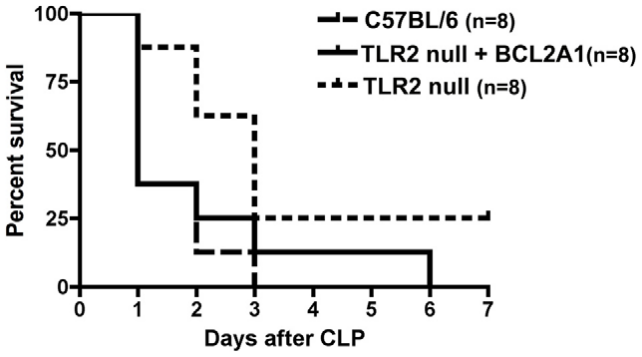
Treatment with rhBCL2A1 does not protect TLR2-null mice. C57BL/6 or TLR2-null mice were subjected to CLP and followed for 7 days. An assessment table was used as a surrogate marker for death, and the animals were euthanized according to approved criteria. Animals were treated by i.p. injection of saline or 1 µg rhBCL2A1 at 18 hours prior to CLP, at the time of CLP, and then every 12 hours for the 3 consecutive days. There was no significant difference in survival between the treatment groups.

Although rhBCL2 and rhBCL2A1 are not direct TLR2 agonists or antagonists *in vitro* [26], we recently demonstrated that protection against ischemia-reperfusion injury of hind limb by rhBCL2A1 required TLR2 and signaling *in vivo* as evidenced by lack of protection in TLR2-null or MyD88-null mice [26]. Consistent with these results in ischemia-reperfusion injury, treatment with rhBCL2A1 did not protect TLR2-null mice following CLP (Fig. 6).

## Discussion

There is compelling experimental evidence that apoptosis is a major contributor to the multiple organ dysfunction in experimental sepsis [5]. Among the genes related to apoptosis, BCL2 and family members are the most extensively investigated [reviewed in [27]]. In multiple studies transgenic mice over-expressing BCL2 in various target cells have decreased apoptosis and improved survival in sepsis [13], [15], [28], [29]. In a recent study we showed that extracellular administration of rhBCL2 or rhBCL2A1 protein provided protection in murine models of hind limb and cardiac ischemia [26]. In the present study, we demonstrate that exogenous administration of rhBCL2 or rhBCL2A1 protein reduces apoptosis in heart, intestine, and peritoneal neutrophils and improves survival following CLP-induced sepsis. We previously showed that BCL2 is released extracellularly with cell necrosis *in vitro* [26]. In this regard, it is notable that Hotchkiss *et al* [30] reported that administration of necrotic cells improved survival in CLP-induced sepsis. Further, since BCL2 and BCL2A1 are expressed on the plasma membrane in some cells [31], [32], it is also possible that BH4-domain proteins are shed during activation or apoptosis as occurs with other plasma membrane proteins. Notably, soluble BCL2 has been detected in extracellular fluids in a variety of clinical conditions [33]-[37]. Thus, BCL2 and BCL2A1 join a growing list of alarmins or DAMPs that modulate the response of the innate immune system to tissue injury [16], [17]. However, in contrast to many other alarmins or DAMPs, BCL2 and BCL2A1 do not appear to elicit a pro-inflammatory response in unstressed animals, either *in vitro* [26] or *in vivo*. Indeed, we analyzed 69 serum proteins following intravenous administration of BCL2A1 protein and found no significant changes from baseline (Table S2).

In preliminary experiments we sought to determine whether treatment following induction of CLP might still be protective. As shown in Fig. S2, treatment at 24 hours after CLP was not effective. However, it is important to note that this is a highly lethal model of sepsis with many animals succumbing within the first 24–48 hours. Consequently, it is likely that many of the animals are already not salvageable by 24 hours. To definitively address the question as to whether post-treatment would be effective would require establishing a less stringent model of sepsis in which the early mortality was not as marked.

Animals treated with rhBCL2- or rhBCL2A1 exhibited reduced numbers of TUNEL-positive cells as well as decreased caspase-3 expression in cardiomyocytes and intestinal cells following CLP, suggesting that improved survival may be due, at least in part, to a reduction of apoptosis in multiple organs. Interestingly, the decreased apoptosis in the rhBCL2A1-treated animals was associated with increased expression of endogenous mouse BCL2 protein in heart and intestinal cells as determined by both immuno-cytochemistry and flow cytometry. Also, there was a significant increase in the total number of neutrophils in the peritoneal lavage fluid from mice receiving rhBCL2A1 followed by CLP, which was likely due to a reduction in neutrophil apoptosis as evidenced by decreased annexin-V staining. This early increase in neutrophils at the site of infection might serve to contain the infection, consequently improving survival. Since murine neutrophils express BCL2A1 but not BCL2, it sees likely that protection by BCL2 or BCL2A1 protein involves up-regulation of other cytoprotective genes (or down-regulation of pro-apoptotic genes).

The endoplasmic reticulum (ER) stress pathway or unfolded protein response (UPR) is one potential target for extracellular BCL2 or BCL2A1 protein [38]. Sepsis [39] and I/R [40] have both been shown to activate the ER stress response and modulation of the response reduces apoptosis in these models. In previous studies we showed that the BH4-domains of BCL2, BCL2A1, and BCL-Xl were protective [26]. Extracellular BCL2 or BCL2A1 protein (via the BH4 domain) could regulate the ER stress response by binding to and signaling through a receptor such as a cell surface Bax inhibitor-1 (BI-1) family member [40]-[42] or membrane voltage–dependent anion channel (VDAC) [43]. BCL2 or BCL2A1 protein could internalize and bind to ER BI-1 or 1,4,5-inositol trisphosphate receptor (I3PR) [44] to suppress an ER stress. BCL2A1 binding to VDAC may also impact ER-triggered mitochondrial membrane permeabilization [45]. In preliminary studies, we have observed uptake of extracellularly administered rhBCL2 protein by neutrophils *in vivo* (Fig. S3, A, B) and by cells *in vitro* (Fig. S3, C-F). Further, we have also demonstrated binding of a biotinylated-BH4-BCL2 peptide to cells *in vitro* (Fig. S4). Future studies should identify the plasma membrane or intracellular target(s) of the extracellularly administered BCL2 proteins.

As previously reported for ischemia-reperfusion injury [26], we found that administration of rhBCL2A1 failed to protect TLR2-null mice from sepsis-induced mortality, indicating that there is a requisite role for TLR2 signaling for the protective effect *in vivo*. However, neither rhBCL2 nor rhBCL2A1 is a direct TLR2 ligand as assessed by IL-8 production in HEK cells transfected with TLR2/6 or by activation of NF-κB in the THP1 macrophage cell line [26]. Also, the response of THP1 macrophage cells to several TLR2 and TLR4 ligands was unaffected by pretreatment with rhBCL2A1 [26]. As discussed previously [26], there are several potential explanations for the discrepancy between the *in vivo* dependence on TLR2 signaling and the lack of direct TLR signaling *in vitro* results with respect to TLR2. First, there may be an intermediate effector cell(s) *in vivo* that elaborates an endogenous TLR2 ligand(s) in response to stimulation by rhBCL2A1 or rhBCL2. Second, BCL2 or BCL2A1 may require a co-factor for activity *in vivo* as has been described for the prototypic DAMP, HMGB1, which lacks significant activity *in vitro* when it is highly purified [23]-[25], but acquires activity upon binding to other TLR ligands [25] or cytokines [24]. Third, since TLR2 heterodimerizes with TLR1 and TLR6 and TLR2 signaling is modulated by several non-TLR receptors [46]-[48], it is possible that signaling in response to rhBCL2 or rhBCL2A1 protein involves a co-receptor and that co-operative signaling by this receptor and TLR2 is responsible for cytoprotection *in vivo*. Fourth, A1 may prime cells for a TLR2 response to oxidized lipoproteins produced during ischemia [49]. Significantly, TLR2 signaling has recently been shown to suppress the ER stress response [50].

In summary, we report that extracellular administration of rhBCL2 or rhBCL2A1 protein provides substantial protection in CLP-induced sepsis in mice. Although the precise cellular and molecular pathways involved in the extracellular activity of BCL2 and BCL2A1 remain to be elucidated, the protective effect is associated with a reduction in apoptosis in the intestine and heart, increased expression of endogenous BCL2 in epithelial cells and cardiomyocytes, and increased neutrophil accumulation in peritoneum. Given that the BCL2 BH4-domain proteins themselves do not appear to elicit a pro-inflammatory response [26](Table S2), as is observed with many other DAMPs, it is possible that extracellular administration of rhBCL2 or rhBCL2A1 protein may offer a new approach to therapy of sepsis.

## Materials and Methods

### Mice

All protocols were approved by the Animal Care and Use Committee of the University of Washington (IACUC #2234-10, 08/05/2009) and complied with the NIH guidelines for care and use of animals. Mice were maintained at the University of Washington, Department of Comparative Medicine facility. C57BL/6 mice were obtained from Charles River Breeding Laboratory (Wilmington, MA). TLR2-null on C57BL/6 background were bred in the animal facility at the University of Washington. Breeding pairs were a gift of Dr. S. Akira (Osaka, Japan).

### Reagents

rhBCL2, rhUbiquitin, rhBim, and rhBCL2A1 were purchased from R&D Systems (Minneapolis, MN). rhBCL2 protein consisted of amino acids 1-212 (accession number P10415) of human BCL2 with the carboxyl terminus replaced with a ten histidine tag. rhBCL2A1 protein consisted of amino acids 1-152 of human BCL2-related protein BCL2A1 (accession number NP004040) missing the carboxyl terminal hydrophobic domain, which was replaced with a six histidine tag. rhUbiquitn consisted of amino acids 1-76 (accession number CAA28495.1) containing the amino acids ATVID followed by a 10 histidine tag at its amino terminal. rhBim consisted of amino acids 2-120 (accession number AAC39594) of human Bim long with a deleted carboxyl terminal transmembrane domain replaced with a six histidine tag. Purity of the proteins was >95% for the recombinant proteins as determined by SDS-PAGE and visualized by silver stain per manufacturer (R&D Systems). In addition, endotoxin concentration in the recombinant proteins was less than 0.15 EU/mg protein (R&D systems).

### Cecal ligation and puncture

Mice were anesthetized with halothane, an abdominal midline incision made and the cecum was gently removed and ligated below the ileocecal valve without obstruction of the ileum or colon. The cecum was then subjected to a single ‘through and through’ perforation with a 20-gauge needle and a small amount of cecal contents gently expressed through the needle wound. The bowel was carefully returned to its original position, and then the abdominal incision was closed in layers with 4-0 sutures.

### Survival after CLP

Mice were followed for up to 7 days after CLP. Each animal was scored using an assessment form that evaluated each animal's health as described by Morton and Griffiths [51], and the animal was euthanized if it exceeded a predetermined score indicative of irreversible sepsis. All animals received 0.5 mg of imipenem in 1.0 ml of 5% dextrose in water 2 hours after CLP. Additional doses of 0.5 mg/mouse of imipenem in 1.0 ml of 5% dextrose in water were given twice a day for 4 days. Mice were treated with rhBCL2A1, rhBCL2, rhBim or rhUbiquitin as indicated.

### TUNEL assay

Mice were sacrificed 24 hours after CLP, and their heart and entire intestine were removed. The intestine was opened along the length of its cephalocaudal axis and washed in 4% neutral-buffered formalin to removed lumenal contents. The tissue was rolled from the proximal to the distal end and fixed with 10% neutral-buffered formalin. Paraffin-embedded sections from heart and intestine were examined for DNA strand breaks by TUNEL (In Situ Cell Death Kit, Roche Molecular Biochemicals) as described by the manufacturer.

### Immunocytochemistry

Tissue sections were pre-treated by boiling in citrate buffer (pH 6.0) in a microwave oven. Immunocytochemistry was accomplished using rabbit anti-mouse BCL2 polyclonal antibody (Upstate, Waltham, MA), or an antibody recognizing active caspase-3 (Cell Signaling, Beverly, MA) using a Vectastain ABC elite kit (Vector Labs, Burlingame, CA), and 3, 3′-diaminobenzidine (Vector Labs) as a chromagenic substrate with hematoxylin counterstaining.

### Quantitation of Images

Images were acquired using a Nikon Labphot microscope with Nikon Plan Fluor 20 Ph3DL (numerical aperture: 0.75) objective (Nikon Instruments, Inc, NY) and Spot v.3.5.9 (Diagnostic Instruments Sterling Heights, MI) digital imaging system. The images from the tissue stained for mBCL2 were opened in Photoshop (Adobe Systems Inc. San Jose, CA), and staining intensity was determined by luminosity of the image with no alteration to the original image.

### Leukocytes and cytokines in peritoneal lavage fluid and peripheral blood

Peritoneal lavage fluid was collected from mice 24 hours after CLP as described previously [15]. The total number of cells and percent of subpopulations in peritoneal lavage fluid were also determined as previously described [15]. The concentration of cytokines in the peritoneal lavage fluid was determined using a mouse cytokine twenty-plex according to manufacturer's protocol (Invitrogen, Multiplex Kit #LMC0006). The numbers of various cell populations in whole blood were determined using a HEMAVET 950 (Drew Scientific Inc., Dallas, TX).

### Assessment of neutrophil viability in peritoneal lavage fluid

Peritoneal neurtophils were collected by lavage, and contaminating fecal debris was removed by filtering through 100 µm filters. Cells were washed with PBS-1%FBS and resuspended in PBS-1%FBS for determination of cell counts. Cell viability was assessed using annexin V apoptosis detection kit (BD Biosciences, San Jose, CA) containing annexin V-PE and. 2×10^5^ cells in 100 µl of binding buffer were incubated with 2 µl of annexin V-PE and 2 µl of 7-amino-actinomycin D for 15 min at 25 °C in the dark. Flow cytometry analysis was performed on a BD FACScan. Live cells were defined as staining negatively for both annexin V and 7-amino-actinomycin D. Flow cytometry data was analyzed with CellQuest software (BD Biosciences). Data are presented as percentage of live cells in each treatment group.

### FACS analysis

We prepared single cell suspensions of cardiomyocytes from hearts harvested 24 hours after CLP as follows. Mice received an injection of heparin (5000 U/kg, ip.) 10 minutes prior to euthansia by exsanguination by cardiac puncture under anesthesia. The animal's chest was opened, heart exposed, and the aorta cannulated with a 24-guage angio-catheter. The heart was perfused with 3 ml of perfusion buffer (AfCS Procedure Protocol PP00000125), followed by perfusion with a digestion buffer (collagenase type II, 75 U/mL, trypsin, 0.14 mg/ml, CaCl2, 1.5 in perfusion buffer) for 20 minutes at 0.5 ml/min. The hearts were placed into a petri dish containing the digestion buffer and manually cut into small pieces. The tissues were transferred to 15 ml tubes in stop buffer (10% BCS, 12.5 µM CaCl2 in perfusion buffer) and further dissociated by triturating using a Pasteur pipette. The samples were centrifuged, the supernatant removed and transferred to a 60 mm petri dish. Calcium was adjusted to a final calcium concentration of 1 mM by adding CaCl_2_. A single cell suspension was prepared by passing the samples through a 40 mm mesh filter. Cells were fixed (BD Cytofix Buffer) for 30 minutes at 4 °C, washed and incubated overnight with the primary antibody to mouse BCL2 or isotype control (BD Pharmingen 556537) at 4 °C. The following day, the cells were washed, resuspended in FACS buffer, and analyzed by FACScan.

### Determination of Reactive Oygen Species generation

Three ml of peritoneal fluid were obtained from each mouse at 24 hours post-CLP. Leukocytes were obtained by centrifugation and lysis of red cells. Equal numbers of leukocytes were loaded with 5 µM DHR123 (Molecular Probes, Eugene, OR) and washed. Cells were resuspended in RPMI medium containing 2% serum. After a15 min incubation at 37 °C with or without 100 ng/ml phorbol myristate acetate (PMA) (Sigma-Aldrich, ST. Louis, MO), cells were removed to ice and fixed with formaldehyde (Polysciences, Inc., Warrington, PA). Flow cytometry data were acquired on a FACScan (Becton-Dickinson, San Jose, CA) and analyzed using CELLQuest software (BD).

### Statistical analysis

The data are presented as the means ± standard error of the mean. Statistical analysis was performed using the two-tailed t-test (GraphPad Prism). Survival curves were analyzed by logrank test (GraphPad Prism). Differences associated with a value of p<0.05 were considered statistically significant.

## Supporting Information

Table S1 Treatment with rhBCL2A1 does not alter cytokine, chemokine, or growth factor levels in peritoneal lavage fluid following CLP.
(0.04 MB DOC)

Table S2 Plasma analytes after injection of rhBCL2A1 in rats
(0.18 MB DOC)

Figure S1 G-CSF levels in spleen and peritoneal fluid following CLP. Mice were treated with 1 µg of rhBCL2A1 or saline on the day prior to and at time of CLP. Spleen and peritoneal lavage fluid were collected at 24 hrs after CLP. G-CSF expression levels were determined in spleen by RT-PCR using primer probes and 7900HT system from Applied Biosystems. Mouse G-CSF protein was determined in the peritoneal fluids using a single analyte ELISA kit (SABiosciences Corporation, Frederick, MD) according to the manufacturer's instructions. *p = 0.049 by unpaired t-test.
(2.19 MB TIF)

Figure S2 Treatment with rhBCLA1 at 24 hours following CLP. Animals were subjected to CLP with no pretreatment. At 24 hours after CLP mice were given 10 µg rhBCLA1 by i.p. injection or saline vehicle control and then treated with 1 µg rhBCL2A1 or saline every twice daily for next 3 days for a total of seven doses.
(6.45 MB TIF)

Figure S3 Uptake of rhBCL2 by rodent cells in vivo and in vitro. In vivo: A rat was treated with 20 µg of rhBCL2 protein given by i.p. injection and at one hour after treatment peripheral blood was collected and leukocytes were isolated following lysis of red cells. Immuno-staining for hBCL2 was performed using an anti-hBCL2 antibody (BD Pharmingen #554231), which does not cross-react with the rodent protein. (A) A preparation of peripheral blood rat leukocytes is shown in low magnification with nuclei counter-stained with DAPI (blue). (B) ∼5% of rat neutrophils showed immuno-staining for hBCL2 protein as shown in the confocal microscopic image (original magnification: ×600). In vitro: JAWSII dendritic cells were incubated with medium containing 2 µg/ml hBCL2 protein for 4 (D) or 24 hours (E, F). In (C) cells were incubated with isotype-control antibody. (D) After four hours incubation prominent immuno-staining for hBCL2 was observed in cytoplasm (original magnification ×400). (E, F) After 24 hours incubation, immuno-staining for hBCL2 was still observed but was not as prominent as at 4 hours. (original magnification: E, ×100; F, ×400).
(4.48 MB TIF)

Figure S4 Binding of BH4-BCL2 peptide to cell surface in vitro. The binding of biotinylated-BH4-BCL2 peptide (SynPep, Dublin CA), scrambled biotinylated-sBH4-BCL2 peptide, and biotinylated-BH3-Bax peptide to JAWS2 dendritic cells grown in (A) suspension or (B) adherent was assessed after incubation for 30 minutes at 4C. Binding was determined by subsequent binding of phycoerythrin-strepavidin and flow cytometry.
(2.33 MB TIF)
