# Supplementary material for: Extracellular Administration of BCL2 Protein Reduces Apoptosis and Improves Survival in a Murine Model of Sepsis
Source: PLoS One. 2011 Feb 24;6(2):e14729. doi: 10.1371/journal.pone.0014729 (PMC3044724; doi:10.1371/journal.pone.0014729)
Supplement: Table S1 — Treatment with rhBCL2A1 does not alter cytokine, chemokine, or growth factor levels in peritoneal lavage fluid following CLP. (0.04 MB DOC) [file pone.0014729.s001.doc]

**Table S1. Treatment with rhBCL2A1 does not alter cytokine, chemokine, or growth factor levels in peritoneal lavage fluid following CLP.**

| Analyte | BCL2A1 (ng/ml ± SD) | Bim (ng/ml ± SD) |
| --- | --- | --- |
| IL-1α | 0.07 ± 0.06 | 0.09 ± 0.10 |
| IL-6 | 0.63 ± 0.90 | 0.50 ± 0.62 |
| IL-10 | 0.29 ± 0.19 | 0.22 ± 0.13 |
| KC | 0.42 ± 0.54 | 0.26 ± 0.24 |
| MCP-1 | 0.46 ± 0.71 | 0.25 ± 0.32 |
| MIP-1α | 0.44 ± 0.72 | 0.37 ± 0.60 |
| FGF-2-basic | 0.14 ± 0.02 | 0.09 ± 0.05 |
| GM-CSF | 0.05 ± 0.01 | 0.05 ± 0.002 |
| VEGF | 0.024 ± 0.01 | 0.04 ± 0.02 |
| TNF-α | 0.05 ± 0.01 | 0.05 ± 0.01 |
| MIG | 0.03 ± 0.002 | 0.04 ± 0.01 |
| IP-10 | 0.12 ± 0.02 | 0.12 ± 0.004 |
| IL-5 | 0.05 ± 0.002 | 0.05 ± 0 |
| IL-4 | 0.04 ± 0.01 | 0.05 ± 0.01 |
| IL-2 | 0.07 ± 0.06 | 0.07 ± 0.03 |
| IL-1α | 0.05 ± 0.08 | 0.04 ± 0.01 |
| IL-17 | 0.04 ±0.01 | 0.03 ± 0 |
| IL-13 | TR | TR |
| IL-12 | 0.01 ± 0.002 | 0.01 ± 0 |
| IFN-γ | 0 | 0 |

TR = trace

Mice were treated once by i.p. injection of 1 μg rhBCL2A1 or rhBim at 18 hours prior to the CLP. At 24 hours following CLP animals were euthanized and peritoneal lavage fluid was collected. The concentration of analytes in the peritoneal lavage fluid was determined using a mouse twenty-plex multiplex assay according to manufacturer’s protocol (Invitrogen, Multiplex Kit #LMC0006). There were no significant differences between levels in the rhBCL2A1- and rhBim-treated animals (n=6 for both groups).
