# Supplementary material for: Extracellular Administration of BCL2 Protein Reduces Apoptosis and Improves Survival in a Murine Model of Sepsis
Source: PLoS One. 2011 Feb 24;6(2):e14729. doi: 10.1371/journal.pone.0014729 (PMC3044724; doi:10.1371/journal.pone.0014729)
Supplement: Table S2 — Plasma analytes after injection of rhBCL2A1 in rats (0.18 MB DOC) [file pone.0014729.s002.doc]

|  |  | **Sampling time after IV injection of rhBCL2A1 protein** | | | | | | | | | |
| --- | --- | --- | --- | --- | --- | --- | --- | --- | --- | --- | --- |
| **Analyte** | units | **0 (Baseline)** | | **2hrs** | | **4hrs** | | **6hrs** | | **24hrs** | |
|  |  |  | SD |  | SD |  | SD |  | SD |  | SD |
| **Apo A1** | ug/mL | **3.2** | 0.7 | **4.67** | 1.57 | **2.30** | 1.58 | **1.9** | 0.4 | **1.2** | 0.8 |
| **Beta-2** | ug/mL | **16.0** | 6.4 | **12.35** | 3.10 | **7.29** | 3.37 | **5.3** | 2.0 | **3.6** | 1.70 |
| **Calbindin** | ng/mL | **0.7** | 1.0 | **0.35** | 0.23 | **0.52** | 0.31 | **0.4** | 0.1 | **0.3** | 0.2 |
| **CD40** | pg/mL | **UDV** | UDV | **UDV** | UDV | **UDV** | UDV | **UDV** | UDV | **UDV** | UDV |
| **CD40 Ligand** | pg/mL | **136.3** | 17.8 | **114.20** | 26.56 | **52.85** | 40.90 | **46.9** | 6.0 | **26.4** | 20.5 |
| **Clusterin** | ug/mL | **99.5** | 17.3 | **93.15** | 24.45 | **44.98** | 32.11 | **38.5** | 6.4 | **22.5** | 16.1 |
| **CRP** | ug/mL | **685.5** | 118.0 | **648.83** | 174.17 | **313.67** | 223.44 | **268.6** | 45.1 | **156.8** | 111.7 |
| **Cystatin-C** | ng/mL | **684.7** | 126.0 | **576.67** | 203.67 | **302.11** | 183.04 | **242.6** | 59.5 | **151.1** | 91.5 |
| **EGF** | pg/mL | **6.6** | 0.6 | **8.39** | 2.34 | **3.76** | 3.08 | **3.4** | 0.3 | **1.9** | 1.5 |
| **Endothelin-1** | pg/mL | **14.2** | 0.0 | **14.20** | 0.00 | **4.73** | 6.31 | **5.5** | 0.8 | **3.2** | 2.4 |
| **Eotaxin** | pg/mL | **1143.3** | 453.3 | **847.33** | 323.56 | **541.41** | 203.95 | **372.7** | 168.7 | **270.7** | 102.0 |
| **Factor VII** | ng/mL | **1.5** | 0.1 | **1.41** | 0.26 | **0.59** | 0.55 | **0.6** | 0.0 | **0.3** | 0.3 |
| **FGF-9** | ng/mL | **2.9** | 3.8 | **0.56** | 0.27 | **1.55** | 1.51 | **1.5** | 0.0 | **0.8** | 0.8 |
| **FGF-basic** | ng/mL | **2.0** | 0.1 | **2.08** | 0.19 | **0.79** | 0.86 | **0.8** | 0.0 | **0.4** | 0.4 |
| **Fibrinogen** | ug/mL | **722.0** | 255.3 | **687.50** | 222.00 | **388.28** | 199.48 | **293.9** | 94.4 | **194.1** | 99.7 |
| **GCP-2** | ng/mL | **0.2** | 0.1 | **0.16** | 0.05 | **0.10** | 0.04 | **0.1** | 0.0 | **0.0** | 0.0 |
| **GM-CSF** | pg/mL | **UDV** | UDV | **UDV** | UDV | **UDV** | UDV | **UDV** | UDV | **UDV** | UDV |
| **GH** | ng/mL | **42.9** | 31.5 | **10.14** | 13.29 | **18.32** | 8.81 | **13.6** | 4.8 | **9.2** | 4.4 |
| **GST-alpha** | ng/mL | **1.3** | 1.3 | **1.49** | 1.35 | **1.39** | 0.07 | **0.7** | 0.7 | **0.7** | 0.0 |
| **GST-Mu** | ng/mL | **344.8** | 190.2 | **175.36** | 89.71 | **151.75** | 41.36 | **96.6** | 55.2 | **75.9** | 20.7 |
| **Haptoglobin** | ug/mL | **292.5** | 89.7 | **215.62** | 114.72 | **140.00** | 50.41 | **95.2** | 44.8 | **70.0** | 25.2 |
| **IFN-gamma** | pg/mL | **UDV** | UDV | **UDV** | UDV | **UDV** | UDV | **16.1** | 0 | **7.42** | 0 |
| **IgA** | ug/mL | **2.3** | 0.7 | **2.43** | 0.12 | **1.08** | 0.90 | **1.0** | 0.1 | **0.5** | 0.4 |
| **IL-10** | pg/mL | **335.0** | 43.3 | **344.50** | 32.33 | **140.06** | 136.30 | **138.2** | 1.9 | **70.0** | 68.1 |
| **IL-11** | pg/mL | **233.7** | 141.3 | **208.92** | 91.75 | **147.33** | 41.06 | **94.2** | 53.1 | **73.7** | 20.5 |
| **IL-12p70** | ng/mL | **0.1** | 0.0 | **0.07** | 0.01 | **0.03** | 0.03 | **0.0** | 0.0 | **0.0** | 0.0 |
| **IL-17** | ng/mL | **0.0** | 0.0 | **0.01** | 0.00 | **0.01** | 0.00 | **0.0** | 0.0 | **0.0** | 0.0 |
| **IL-18** | ng/mL | **0.4** | 0.0 | **UDV** | UDV | **UDV** | UDV | **UDV** | UDV | **UDV** | UDV |
| **IL-1alpha** | pg/mL | **97.8** | 105.3 | **68.07** | 49.29 | **74.21** | 20.71 | **47.5** | 26.8 | **37.1** | 10.4 |
| **IL-1beta** | ng/mL | **1.1** | 0.3 | **0.89** | 0.19 | **0.47** | 0.28 | **0.4** | 0.1 | **0.2** | 0.1 |
| **IL-2** | pg/mL | **169.3** | 230.4 | **22.18** | 8.15 | **86.90** | 95.64 | **91.3** | 4.4 | **47.8** | 43.4 |
| **IL-3** | pg/mL | **UDV** | UDV | **UDV** | UDV | **UDV** | UDV | **UDV** | UDV | **UDV** | UDV |
| **IL-4** | pg/mL | **21.2** | 4.0 | **24.80** | 4.40 | **11.07** | 9.16 | **10.1** | 1.0 | **5.5** | 4.6 |
| **IL-5** | ng/mL | **UDV** | UDV | **UDV** | UDV | **UDV** | UDV | **UDV** | UDV | **UDV** | UDV |
| **IL-6** | pg/mL | **13.6** | 0.0 | **4.46** | 0.34 | **1.60** | 1.91 | **1.8** | 0.2 | **1.0** | 0.8 |
| **IL-7** | ng/mL | **0.1** | 0.1 | **0.11** | 0.03 | **0.07** | 0.03 | **0.0** | 0.0 | **0.0** | 0.0 |
| **Insulin** | U/mL | **5.4** | 0.5 | **11.13** | 6.62 | **6.07** | 3.74 | **4.9** | 1.2 | **3.0** | 1.9 |
| **IP-10** | pg/mL | **79.6** | 82.8 | **37.68** | 15.24 | **45.24** | 25.03 | **35.1** | 10.1 | **22.6** | 12.5 |
| **KC/GROalpha** | ng/mL | **0.0** | 0.0 | **0.35** | 0.36 | **0.24** | 0.16 | **0.2** | 0.0 | **0.1** | 0.1 |
| **Leptin** | ng/mL | **1.9** | 1.0 | **1.37** | 0.77 | **1.05** | 0.22 | **0.6** | 0.4 | **0.5** | 0.1 |
| **LIF** | pg/mL | **225.5** | 27.2 | **244.00** | 26.00 | **99.06** | 96.63 | **97.8** | 1.2 | **49.5** | 48.3 |
| **Lymphotactin** | pg/mL | **13.8** | 4.4 | **13.16** | 4.38 | **7.31** | 3.90 | **5.6** | 1.7 | **3.7** | 1.9 |
| **MCP-1** | pg/mL | **600.5** | 87.2 | **1196.00** | 742.67 | **675.28** | 392.07 | **533.7** | 141.6 | **337.6** | 196.0 |
| **MCP-3** | pg/mL | **394.3** | 72.3 | **757.67** | 408.56 | **412.85** | 229.88 | **321.4** | 91.5 | **206.4** | 114.9 |
| **MCP-5** | pg/mL | **4.1** | 0.0 | **UDV** | UDV | **UDV** | UDV | **UDV** | UDV | **UDV** | UDV |
| **M-CSF** | ng/mL | **0.7** | 0.1 | **0.61** | 0.14 | **0.28** | 0.22 | **0.3** | 0.0 | **0.1** | 0.1 |
| **MDC** | pg/mL | **474.5** | 136.5 | **298.67** | 64.44 | **166.54** | 88.09 | **127.3** | 39.2 | **83.3** | 44.0 |
| **MIP-1alpha** | ng/mL | **0.2** | 0.0 | **0.15** | 0.04 | **0.08** | 0.04 | **0.1** | 0.0 | **0.0** | 0.0 |
| **MIP-1beta** | pg/mL | **103.8** | 32.9 | **159.25** | 57.50 | **83.20** | 50.70 | **67.0** | 16.3 | **41.6** | 25.4 |
| **MIP-1gamma** | ng/mL | **0.0** | 0.0 | **0.01** | 0.00 | **0.00** | 0.00 | **0.0** | 0.0 | **0.0** | 0.0 |
| **MIP-2** | pg/mL | **19.5** | 3.4 | **53.25** | 45.58 | **34.07** | 20.46 | **27.3** | 6.8 | **17.0** | 10.2 |
| **MIP-3beta** | ng/mL | **0.4** | 0.2 | **0.32** | 0.17 | **0.24** | 0.06 | **0.1** | 0.1 | **0.1** | 0.0 |
| **MMP-9** | ng/mL | **UDV** | UDV | **UDV** | UDV | **UDV** | UDV | **UDV** | UDV | **UDV** | UDV |
| **MPO** | ng/mL | **26.3** | 5.0 | **37.25** | 8.77 | **17.01** | 13.49 | **15.3** | 1.8 | **8.5** | 6.7 |
| **Myoglobin** | ng/mL | **615.9** | 729.4 | **748.80** | 884.13 | **787.45** | 64.46 | **426.0** | 361.5 | **393.7** | 32.2 |
| **NGAL** | ng/mL | **147.0** | 42.3 | **156.05** | 71.63 | **90.01** | 44.03 | **67.0** | 23.0 | **45.0** | 22.0 |
| **OSM** | ng/mL | **0.7** | 0.8 | **0.23** | 0.04 | **0.34** | 0.28 | **0.3** | 0.0 | **0.2** | 0.1 |
| **Osteopontin** | ng/mL | **20.9** | 4.5 | **28.94** | 6.27 | **13.23** | 10.47 | **11.9** | 1.4 | **6.6** | 5.2 |
| **RANTES** | pg/mL | **17.3** | 8.0 | **10.04** | 3.83 | **7.30** | 2.31 | **4.8** | 2.5 | **3.6** | 1.2 |
| **SAP** | ug/mL | **30.3** | 3.9 | **28.05** | 4.50 | **12.15** | 10.60 | **11.4** | 0.8 | **6.1** | 5.3 |
| **SCF** | pg/mL | **2233.0** | 1949.0 | **635.50** | 135.00 | **906.50** | 695.00 | **800.8** | 105.8 | **453.3** | 347.5 |
| **SGOT** | ug/mL | **UDV** | UDV | **UDV** | UDV | **UDV** | UDV | **UDV** | UDV | **UDV** | UDV |
| **TIMP-1** | ng/mL | **4.9** | 0.8 | **5.60** | 0.84 | **2.41** | 2.13 | **2.3** | 0.1 | **1.2** | 1.1 |
| **Tissue Factor** | ng/mL | **2.3** | 0.4 | **2.32** | 0.30 | **1.01** | 0.87 | **0.9** | 0.1 | **0.5** | 0.4 |
| **TNF-alpha** | ng/mL | **0.1** | 0.0 | **0.10** | 0.04 | **0.05** | 0.03 | **0.0** | 0.0 | **0.0** | 0.0 |
| **TPO** | ng/mL | **3.5** | 0.0 | **UDV** | UDV | **UDV** | UDV | **UDV** | UDV | **UDV** | UDV |
| **VCAM-1** | ng/mL | **143.3** | 9.4 | **125.08** | 31.22 | **55.25** | 46.56 | **50.9** | 4.3 | **27.6** | 23.3 |
| **VEGF** | pg/mL | **272.3** | 67.8 | **380.00** | 40.67 | **162.8** | 144.7 | **153.** | 9.0 | **81.4** | 72.4 |
| **vWF** | ng/mL | **284.3** | 86.3 | **283.52** | 91.64 | **153.8** | 86.46 | **120.** | 33.7 | **76.9** | 43.2 |

**Table S2. Plasma analytes after injection of rhBCL2A1 in rats.**

Multiplex analysis was performed at Rules-Based Medicine (Austin, TX), which uses multi-analyte profiles (MAPs) based on Luminex xMAP® (Luminex Corporation, Austin TX) technology. A total of 69 analytes were analyzed. Carotid arteries of 6 rats were isolated and a catheter placed for blood sampling. 0.5 ml of blood was withdrawn through the catheter and replaced with 0.5 ml of saline at each time-point. At time zero, after baseline sampling, 20 μg of rhBCL2A1 protein was given by i.p. injection. Samples were withdrawn at 2,4, and 6 hrs after treatment. At 24 hrs post-injection, animals were euthanized and 8-10 ml of blood was taken for analysis. UDV= undetectable value
