# Supplementary figures and images for: Extracellular Administration of BCL2 Protein Reduces Apoptosis and Improves Survival in a Murine Model of Sepsis
Source: PLoS One. 2011 Feb 24;6(2):e14729. doi: 10.1371/journal.pone.0014729 (PMC3044724; doi:10.1371/journal.pone.0014729)

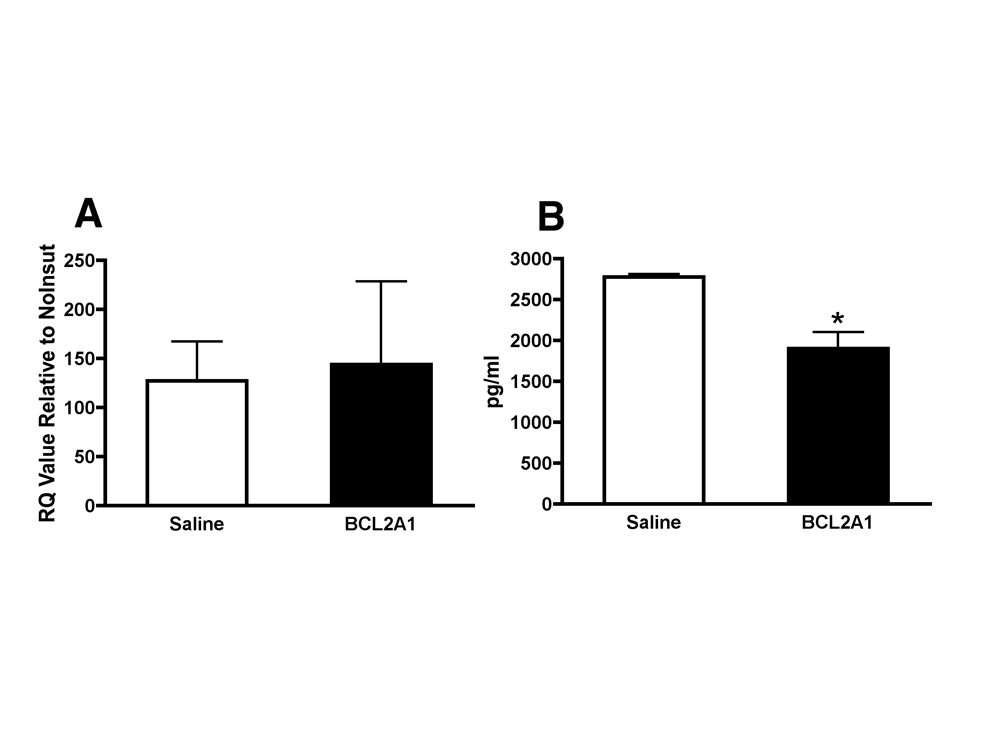

Supplement: Figure S1 — G-CSF levels in spleen and peritoneal fluid following CLP. Mice were treated with 1 µg of rhBCL2A1 or saline on the day prior to and at time of CLP. Spleen and peritoneal lavage fluid were collected at 24 hrs after CLP. G-CSF expression levels were determined in spleen by RT-PCR using primer probes and 7900HT system from Applied Biosystems. Mouse G-CSF protein was determined in the peritoneal fluids using a single analyte ELISA kit (SABiosciences Corporation, Frederick, MD) according to the manufacturer's instructions. *p = 0.049 by unpaired t-test. (2.19 MB TIF) [file pone.0014729.s003.tif]

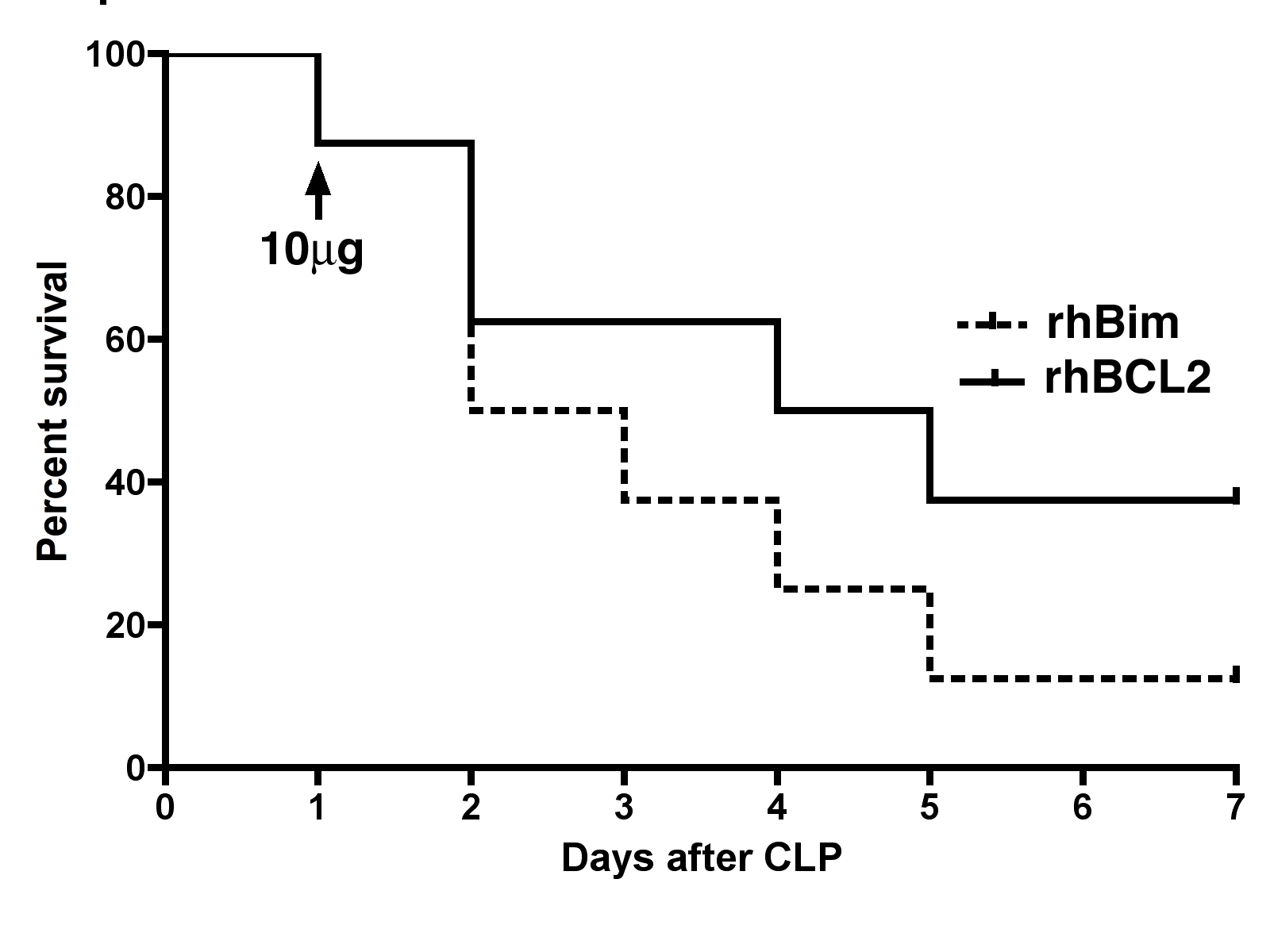

Supplement: Figure S2 — Treatment with rhBCLA1 at 24 hours following CLP. Animals were subjected to CLP with no pretreatment. At 24 hours after CLP mice were given 10 µg rhBCLA1 by i.p. injection or saline vehicle control and then treated with 1 µg rhBCL2A1 or saline every twice daily for next 3 days for a total of seven doses. (6.45 MB TIF) [file pone.0014729.s004.tif]

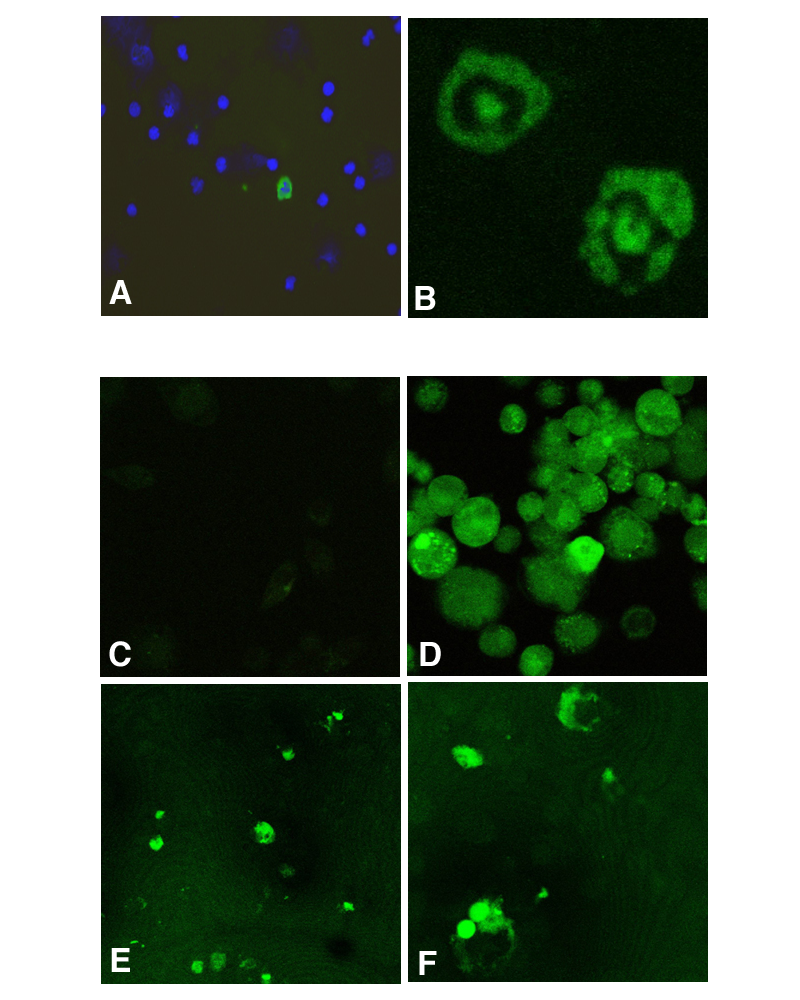

Supplement: Figure S3 — Uptake of rhBCL2 by rodent cells in vivo and in vitro. In vivo: A rat was treated with 20 µg of rhBCL2 protein given by i.p. injection and at one hour after treatment peripheral blood was collected and leukocytes were isolated following lysis of red cells. Immuno-staining for hBCL2 was performed using an anti-hBCL2 antibody (BD Pharmingen #554231), which does not cross-react with the rodent protein. (A) A preparation of peripheral blood rat leukocytes is shown in low magnification with nuclei counter-stained with DAPI (blue). (B) ∼5% of rat neutrophils showed immuno-staining for hBCL2 protein as shown in the confocal microscopic image (original magnification: ×600). In vitro: JAWSII dendritic cells were incubated with medium containing 2 µg/ml hBCL2 protein for 4 (D) or 24 hours (E, F). In (C) cells were incubated with isotype-control antibody. (D) After four hours incubation prominent immuno-staining for hBCL2 was observed in cytoplasm (original magnification ×400). (E, F) After 24 hours incubation, immuno-staining for hBCL2 was still observed but was not as prominent as at 4 hours. (original magnification: E, ×100; F, ×400). (4.48 MB TIF) [file pone.0014729.s005.tif]

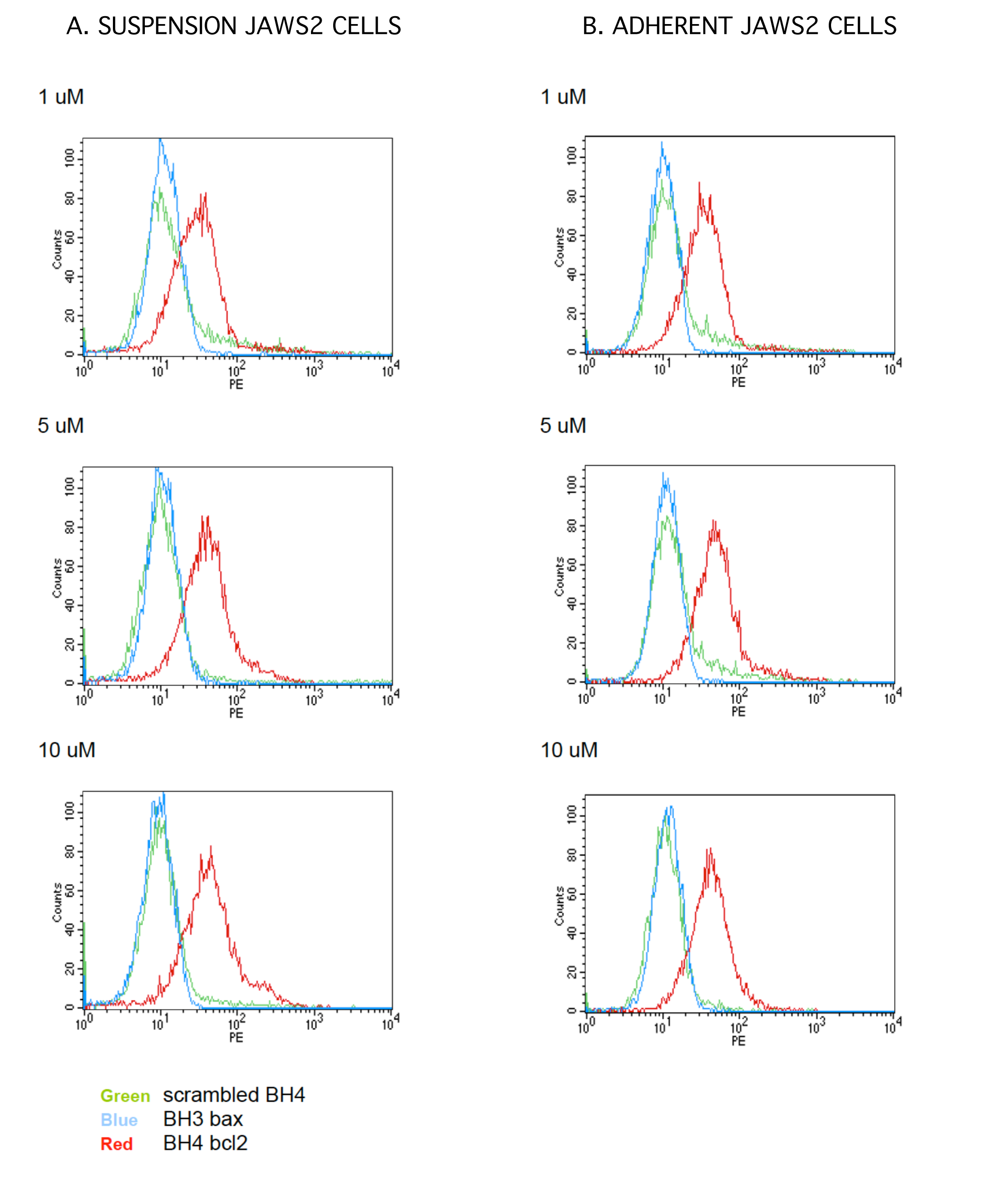

Supplement: Figure S4 — Binding of BH4-BCL2 peptide to cell surface in vitro. The binding of biotinylated-BH4-BCL2 peptide (SynPep, Dublin CA), scrambled biotinylated-sBH4-BCL2 peptide, and biotinylated-BH3-Bax peptide to JAWS2 dendritic cells grown in (A) suspension or (B) adherent was assessed after incubation for 30 minutes at 4C. Binding was determined by subsequent binding of phycoerythrin-strepavidin and flow cytometry. (2.33 MB TIF) [file pone.0014729.s006.tif]
